# Supplementary material for: Asian monsoon rainfall variation during the Pliocene forced by global temperature change
Source: Nat Commun. 2019 Nov 21;10:5272. doi: 10.1038/s41467-019-13338-4 (PMC6872654; doi:10.1038/s41467-019-13338-4)
Supplement: Supplementary file 1 — Supplementary Information [file 41467_2019_13338_MOESM1_ESM.pdf]

Supplementary Information

Asian monsoon rainfall variation during the Pliocene forced by global temperature change

Wang et al

Supplementary Notes

Supplementary Note 1: Modification of conventional phytolith morphotype classification.

Differences in regional vegetation result in differences in the prevailing phytolith morphotypes. Some of these morphotypes are not well classified, which could affect the accuracy of the estimation of C_4 biomass. Therefore, we made modifications to the conventional classification. Phytolith morphotypes from Oryzoideae have been well studied in China[1,2] because rice (*Oryza sativa*) is an important economic species. All the studies indicate that *Oryza* generates a type of biolobate that can be distinguished from others, such as the *Stipa*-type bilobate from Pooideae (C_3) and the panicoid biolobate from Panicoideae (C_4). A systematic study of Oryzoideae phytoliths shows that the scooped biolobate is mainly generated by the BO clade (Bambusoideae-Oryzoideae)[3,4]. In this study, we added scooped biolobate as a characteristic morphotype of the BO clade (Supplementary Table 1).

Supplementary Note 2: Global species-climate dataset

The method we used mainly follows Edwards and Smith[5]. We extracted all geo-referenced natural history collections of Poaceae species that are available via the Global Biodiversity Information Facility (GBIF) web portal [http://www.gbif.org/]. For each set of coordinates, we extracted monthly temperature and precipitation values from the CliMond database [https://www.climond.org/] at 10′ resolution (~18 km at the

equator)[6]. The original collection data consisted of 17,987,291 records. After screening empty (no coordinates/genus/species data) and duplicate records, we obtained a dataset consisting of 9,979,097 independent collections spanning 751 genera and 9921 species. We excluded all hybrid species/genera and species with fewer than 10 independent localities, reducing the dataset to 9,961,948 collections spanning 567 genera and 6018 species. The mean number of collections per species is 1655; this value was heavily influenced by several species (e.g., *Holcus lanatus*, *Dactylis glomerata*, *Festuca rubra*, *Agrostis stolonifera*, *Poa trivialis*) with a large number of collections. After the 5% most heavily collected species were removed, the mean number of collections per species was 257. After matching with climatic data, the final database consisted of 9,390,524 collections including 567 genera and 6010 species with corresponding MAT, WMMT, CMMT, MAP, WMMP, CMMP and difference in temperature of warmest and coldest months (DT). The phylogenetic classification of the Poaceae follows the latest molecular and morphological studies[7,8].

For each species, our dataset could capture its ecological amplitude, optimum range or just a random range inside its ecological amplitude. However, the climate range of each subfamily calculated from the combination of climate ranges of all its species was too wide, and this calculated climate range could approach a subfamily's tolerance limit or exceed its ecological amplitude. For example, Bambusoideae has a lower limit of MAT=1°C and MAP=40 mm, obviously no Bambusoideae could survive this environment. There are several possible explanations for this problem: firstly, the large collection dataset (~10 million) results in a large climate range that approaches each

species' ecological amplitude; secondly, there are some flaws in collection data due to the introduction of species that grow under human care, such as *Triticum aestivum*; thirdly, Landforms could also bias the results by changing the local environment.

For the above reasons, it is inappropriate to use the climate range of each species, which approaches the tolerance limit or exceeds the ecological amplitude to calculate the climate range of each subfamily. The optimum range of each species in which species can grow and reproduce is the best choice but is however difficult to obtain. To simplify the problem, here we make an assumption: for each species, the mean value of its climate range is within its optimum range, and we use this value as the optimum value (e.g., *Achnatherum bromoides* had 497 collections, the optimum MAT of *Achnatherum bromoides* is the mean value of MAT of its 497 collection localities). This value is prone to inherent biases in sampling, human activity, and the competition between species and landform could also bias the value. It is important to note that the climate range of each subfamily calculated by this method ranges from the lowest optimum value of its species to the highest, and the actual climate range is larger.

Environmental niche of global grasses. Climate data extracted from all available geo-referenced grasses provided clear evidence that certain grass lineages have specialized in certain habitats. For temperature, Pooideae and Danthonioideae stand out and occupy a very cool environment, as suggested by MAT, WMMT, and CMMT. Pooideae also stand out and are able to tolerate the most dramatic temperature changes, while Bambusoideae prefer more stable environments. For precipitation, Bambusoideae stand apart as inhabiting the wettest environments of all subfamilies, whereas the open habitat

grasses Panicoideae and Micrairoideae and the closed habitat grasses Oryzoideae occupy humid environments, as suggested by MAP and WMMP. Pooideae occupy the dry end of the spectrum alongside Aristidoideae and Chloridoieae. For seasonal changes in precipitation, Danthonioideae prefer a dry summer and a mild winter; Pooideae, Oryzoideae and Arundinoideae have no preference; and other grasses prefer rain and warmth during the same period. The climate range of each subfamily of Poaceae is too wide for quantitative reconstruction (Supplementary Table 4).

Supplementary Note 3: Data sources for Figure 4

f: Synthesis of pollen and sedimentary records from northwestern China to illustrate the evolution of arid/monsoon climate in East Asia. Data sources: pollen data for Linxia Basin[9], pollen data from Shikouzi in Ningxia[10], pollen data from the Tarim Basin[11], aeolian silt and stratigraphic data from the Guanzhong Basin[12,13]. **h**: Synthesis of published pCO_2 proxy data obtained through a variety of different methodologies. The source for alkenone-based palaeo-CO_2 estimates is blue circles[14-17]; for the boron-based approach, the orange diamonds[14,18-20]; for the B/Ca ratio-based approach, the brown squares[21]; for the stomatal density-based approach, the green circles; and for the palaeosol $\delta^{13}C$ method, dark blue symbols, integrated by Beerling and Royer[22].

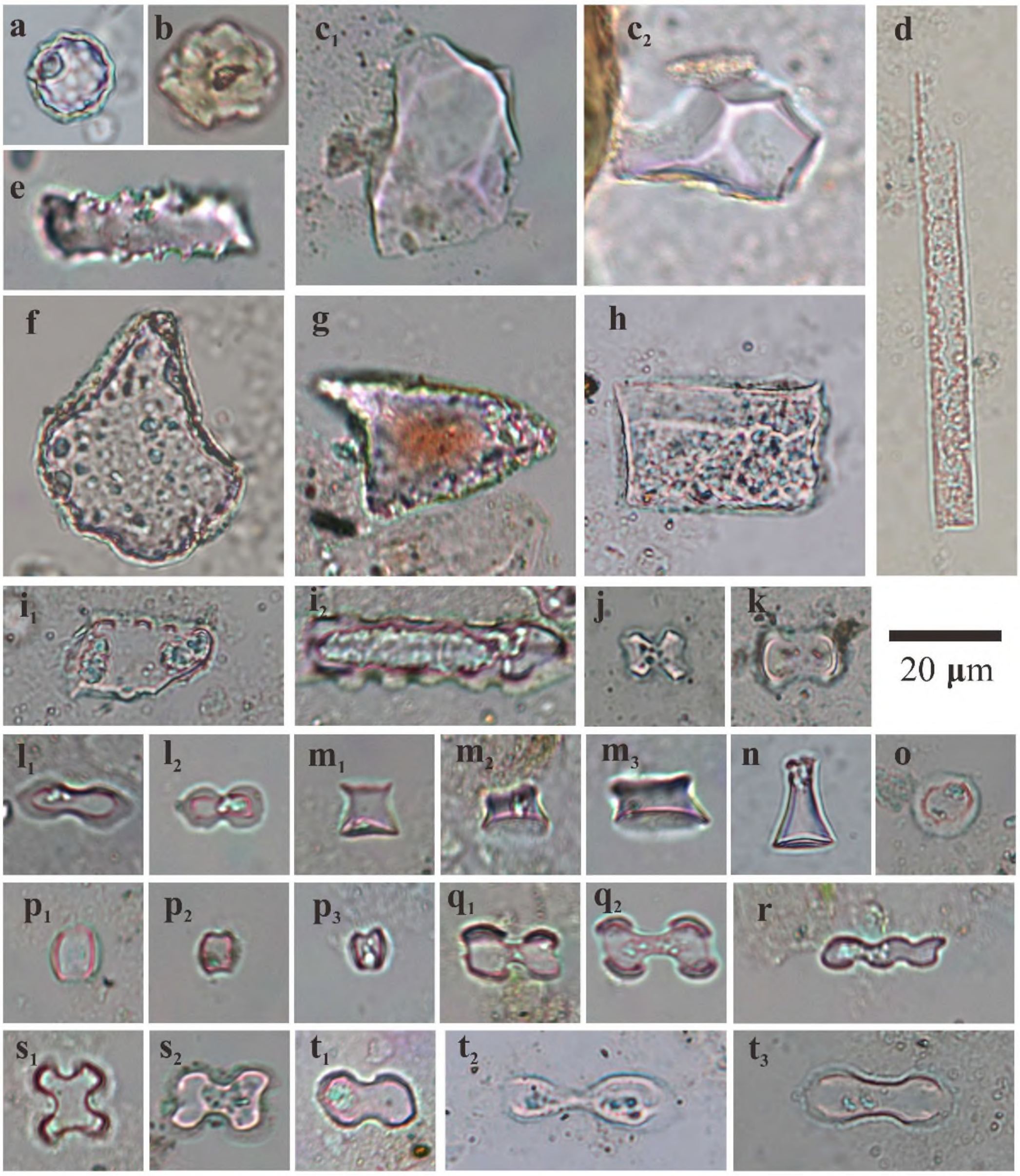

Supplementary Figure 1. Representative phytolith morphotypes found in the Bahe Formation, Lantian Formation and loess-paleosol deposits from the Duanjiapo section, Lantian, central China. **a**, Globular echinate (palm); **b**, globular verrucate (FI); **c**, blocky polyhedron (FI); **d**, elongate psilate (NDO); **e**, elongate echinate (NDG); **f**, cuneiform bulliform cell (NDG); **g**, acicular hair cell (NDG); **h**, parallepipedal bulliform cell (NDG); **i**, trapeziform sinuate (POOID-D); **j**, scooped bilobate (Oryzoideae); **k**, collapsed saddle? (Bambusoideae); **l**,

stipa-type bilobate (POOID-D); **m**, keeled rondel (POOID-ND); **n**, conical rondel (POOID-ND); **o**, trapeziform short cell (POOID-ND); **p**, saddle (CHLOR); **q**, panicoid bilobate (PAN); **r**, cylindrical polylobate (PAN); **s**, cross (PAN); **t**, simple lobate (PACMAD-general). Sample numbers and the stratigraphic profiles for the imaged phytoliths: **a**, Q4-140, Loess; **b**, 2#SWC-650, Lantian Formation; $\mathbf{c}_1$, $\mathbf{c}_2$, 5#SWC-7350, Bahe Formation; **d**, 5#SWC-7650, Bahe Formation; **e**, 5#SWC-7850, Bahe Formation; **f**, 5#SWC-8650, Bahe Formation; **g**, 3#SWC-650, Lantian Formation; **h**, 5#SWC-7750, Bahe Formation; $\mathbf{i}_1$, 5#7350, Bahe Formation; $\mathbf{i}_2$, 5#SWC-7650, Bahe Formation; **j**, 5#SWC-7350, Bahe Formation; **k**, 5#SWC-7650, Bahe Formation; $\mathbf{l}_1$, 5#SWC-7850, Bahe Formation; $\mathbf{l}_2$, 5#SWC-7850, Bahe Formation; $\mathbf{m}_1$, 5#SWC-7850, Bahe Formation; $\mathbf{m}_2$, $\mathbf{m}_3$, 5#SWC-7650, Bahe Formation; **n,** 5#SWC-7850, Bahe Formation; **o,** 5#SWC-7350, Bahe Formation; $\mathbf{p}_1$, 3#SWC-2950, Lantian Formation; $\mathbf{p}_2$, 3#SWC-3950, Lantian Formation; $\mathbf{p}_3$, 5#SWC-7650, Bahe Formation; $\mathbf{q}_1$, $\mathbf{q}_2$, 5#SWC-7750, Bahe Formation; **r**, 5#SWC-7750, Bahe Formation; $\mathbf{s}_1$, 5#SWC-7750, Bahe Formation; $\mathbf{s}_2$, 5#SWC-7850, Bahe Formation; $\mathbf{t}_1$, 3#SWC-2650, Lantian Formation; $\mathbf{t}_2$, 5#SWC-7750, Bahe Formation; $\mathbf{t}_3$, 5#SWC-7650, Bahe Formation.

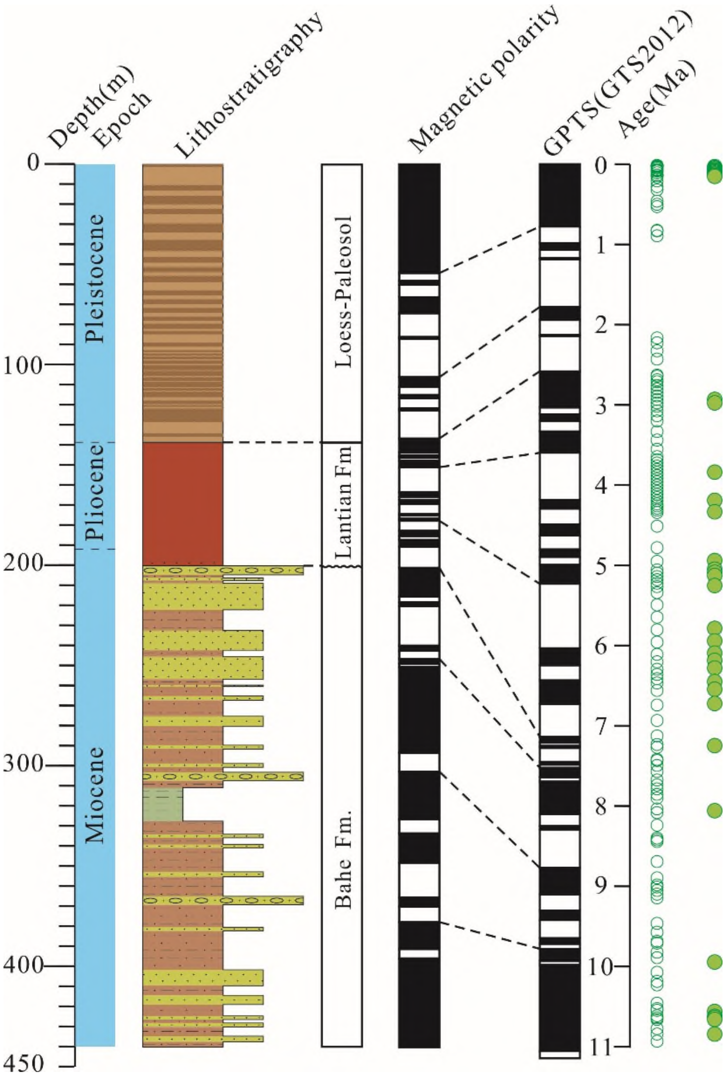

Supplementary Figure 2. Lithostratigraphy and magnetostratigraphy of the Duanjiapo section at Lantian and comparison with the Geomagnetic Polarity Time Scale (GPTS2012)[23]. The palaeomagnetic data between 0-2.6 Ma are after Zheng *et al.*[24], 2.6-7 Ma after An *et al.*[25] and 7-11 Ma after Wang *et al.*[26]. Green open circles: 133 samples collected for phytolith analysis in this study. Green circles: 38 samples with fair to very good phytolith preservation.

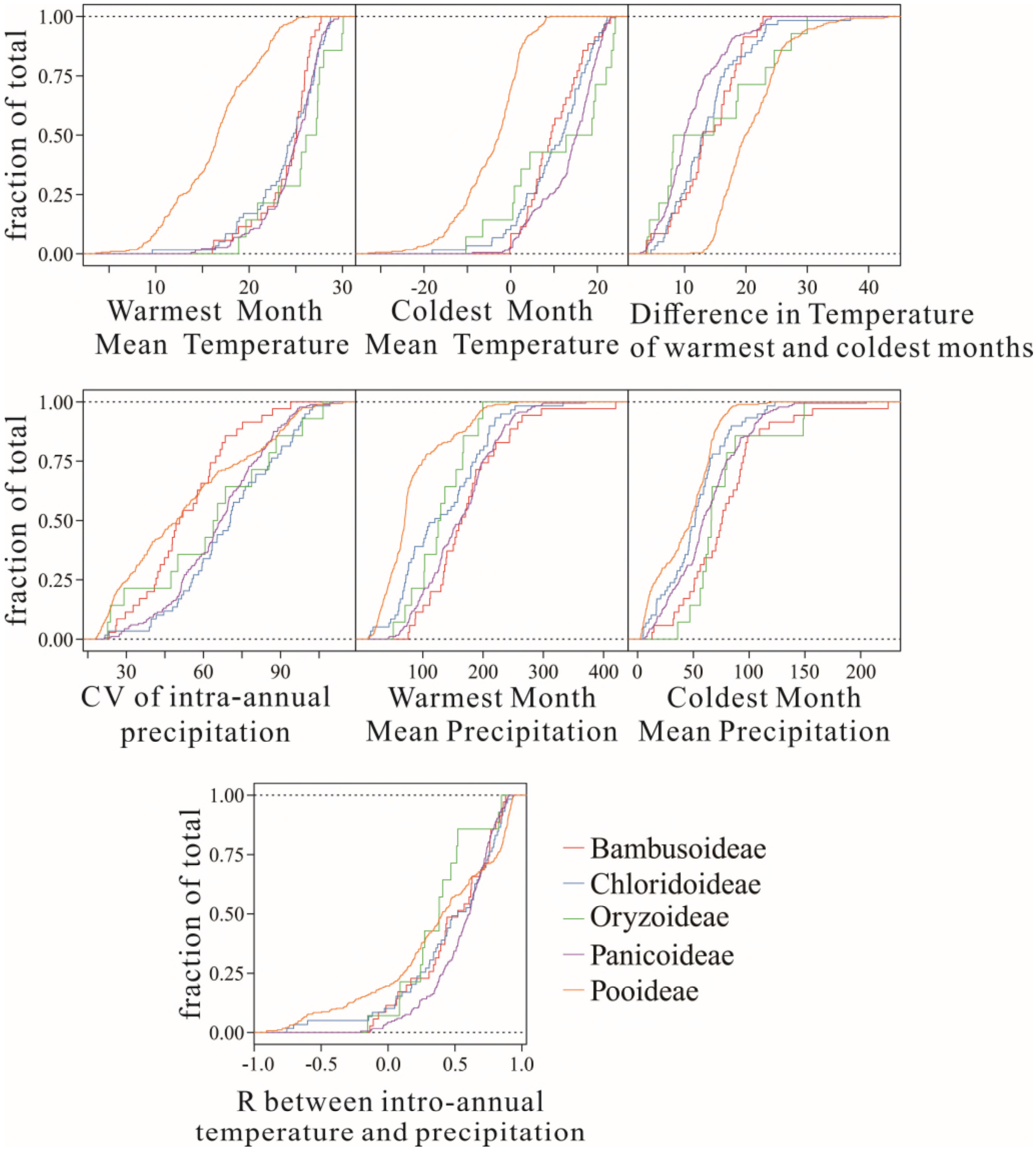

Supplementary Figure 3. Species accumulation curves for the climate parameters in China, sorted by the major grass subfamilies. These data represent 598 species record in Flora of China [http://frps.iplant.cn/] while the collection localities not necessarily in China.

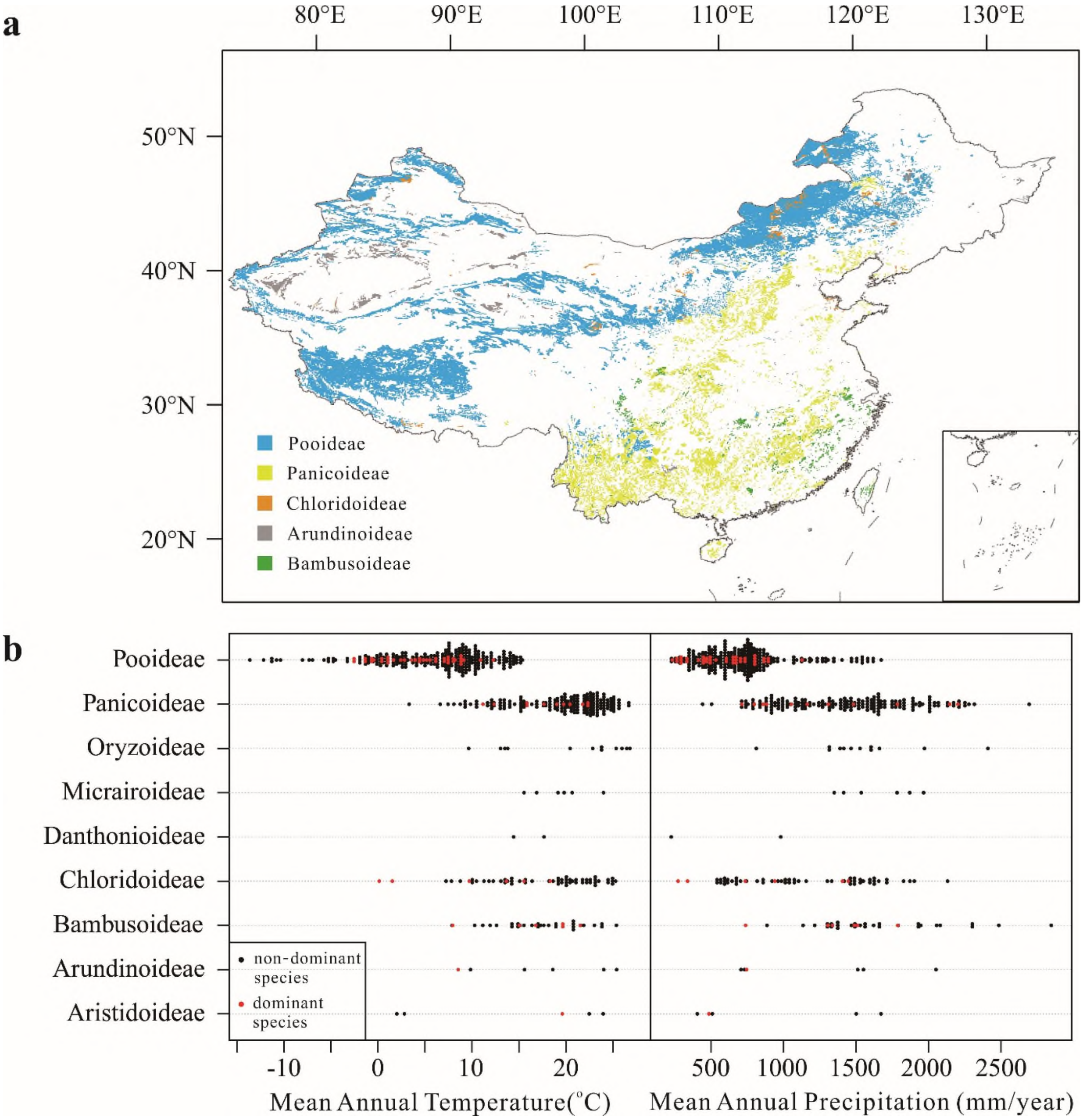

Supplementary Figure 4. Distribution and habitat of grasslands in China. a, Distribution of ecosystem dominated by Poaceae grasses, sorted by major grass subfamilies. **b**, Wilkinson dot plot for mean annual temperature and precipitation of 598 species in China, sorted by major grass subfamilies. Red dot indicates species that dominant grassland according to ecosystem data of China [http://www.ecosystem.csdb.cn].

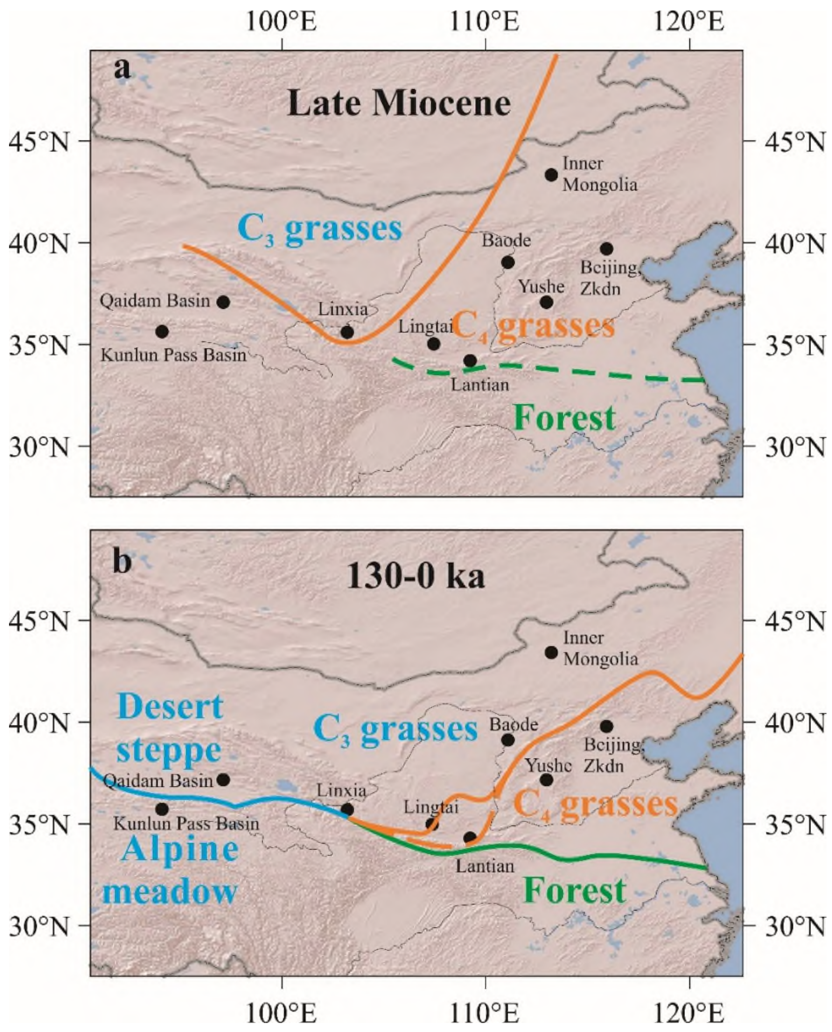

Supplementary Figure 5. Vegetation distribution and its variation since the late Miocene in East Asia. Black dots are locations referenced in Fig. 1. **a-b**, Green line: the boundary between forest and grasses (C_3/C_4) (dashed line indicates an uncertainty in the distribution). Orange line: the boundary between C_3 grasses and C_4 grasses. In **b**, the solid line indicates the distribution of modern vegetation and the dashed line indicates the vegetation distribution inferred from the fossil records (this study). Blue line: the boundary between modern desert steppe and alpine meadow. Data source: The boundary between forest and grassland is mainly based on pollen and fossil mammal data[27,28]. The distribution of C_4 grasses at the

following localities is well studied and can be used to constrain the inferred vegetation distributions. The data are from the Kunlun Pass Basin[29], Qaidam Basin[30], Linxia[31], Lingtai[32,33], Lantian (this study), Baode, Yushe[34], Inner Mongolia[35] and Zhoukoudian[36]. The distribution of modern vegetation is from Chinese terrestrial ecosystem database [http://www.ecosystem.csdb.cn]. The images of landform are from [https://maps.ngdc.noaa.gov/arcgis/rest/services].

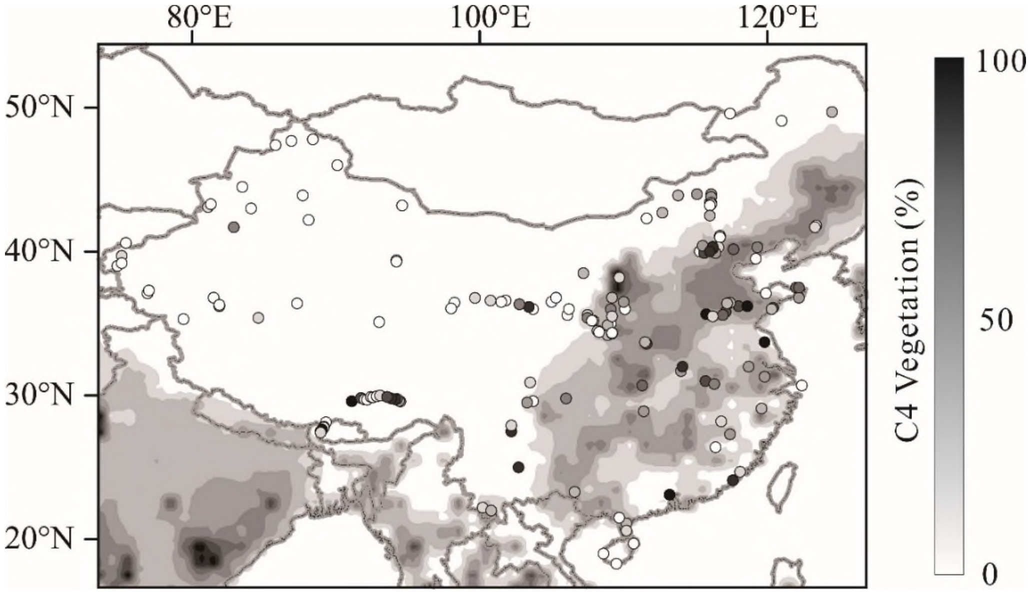

Supplementary Figure 6. A comparison of C_4 percentages estimated by phytolith assemblages (point) and the distribution of modern C_4 plants in Asia (area). The distribution and percentage of modern C_4 plants are after Still *et al.*[37]. For the use of phytolith assemblages to estimate C_4 percentages, we calculated the percentage of C_4 grasses from modern soil phytoliths assemblage data[38] using our method. We chose the samples with the percentage of FI+GSSC> 25% of the total phytolith count which comprises at least 75-125 grains of diagnostic phytoliths.

Supplementary Table 1. Phytolith classification used herein, following Strömberg and McInerney[39] with one modification.

Plant group			Phytolith class	Phytolith morphotypes
Forest indicator taxa (FI)	Palm		FI TOT	Globular echinate*
	Other FI: woody/ hebaceous basal angiosperms and eudicotyledons, conifers,ferns etc.			Globular granulate* (3–10 mm), globular verrucate (>15 mm), simple and compound globular psilate (3–15 mm), polyhedral epidermis (various types), anticlinal epidermis, silicifed nongrass (reniform) guard and subsidiary cells, globular and variously shaped laminar vesicular infilling, irregular sulcate or facetate vascular cell [including Magnolia-type terminal tracheid and tuberculate rounded sclereid/tracheid, favose aggregate, sclerenchyma and similar (irregular clavate body, often branched, sometimes facetate or papillate surface), blocky polyhedron (equidimensional irregular or facetate body).
Poaceae (GSSC)	Closed-habitat grasses (basal Poaceae+BO clade)		CH TOT	*Chusquoid* body/cross with irregular/spiked top,*Chusquea*-type rondel, *Chusquea*-type bilobate, collapsed saddle, regular chusquoid body, crescentic keeled rondel, **scooped bilobate**
	Pooideae	Diagnostic	POOID-D	trapeziform polylobate*, trapeziform sinuate*,Stipa-type Bilobate
		Non-Diagnostic	POOID-ND	Conical and keeled rondel*, trapeziform short cell*, pyramidal
	PACMAD clade	Panicoideae (~C_4)	PAN	Panicoid bilobate*,Cylindrical polylobate*, various crosses*， simple lobate

		Chloridoideae(~C_4)	CHLOR	Saddle*, saddle-like
		PACMAD general (C_3/C_4)	PACMAD general	Inverted bilobate/cross, near-panicoid bilobate, near-panicoid cross, *Merxmuellera*-type rondel (convex/concave base with bulging sides in side view and flat top)
	Unknown Poaceae		OTHG	Various unknown or unidentifiable GSSC
			NDG	Cuneiform/Parallepipedal bulliform cell*,short unciform prickle,"scutiform opal"
Wetland plants	Sedges, Equisetum, unknown aquatic monocotyledon		AQ	papillae*, papillate epidermis, silicified stellate facetate parenchyma
Grasses and other monocotyledons, conifers			NDG	Elongate sinuous, echinate*, and dendritic long cell*, acicular hair cell*, silicified monocotyledon stomatal complex, monocotyledon tracheary element (cylindric sulcate tracheid*), etc.
Various plants and unknown			NDO	Elongate psilate, cylindric psilate, elongate facetate, cylindric papillate, various unknown and unidentifiable morphotypes

FI = forest indicator taxa

GSSC = grass silica short cell produced exclusively by grasses (Poaceae)

BO clade = Bambusoideae + Oryzoideae

PACMAD clade = Panicoideae, Arundinoideae, Chloridoideae, Micrairoideae, Aristidoideae, and Danthonioideae

FI TOT = sum of forest indicator phytoliths

CH TOT = morphotypes typical of closed-habitat grasses in the BO clade, and a variety of basal grasses

POOID-D (Pooideae, diagnostic) = GSSC morphotypes that are diagnostic of grasses in the Pooideae
POOID-ND (Pooideae, non-diagnostic) = GSSC morphotypes that are produced in high frequencies by many grasses in the Pooideae,but that are also found in other grasses
PAN = forms typical of Panicoideae (predominantly C_4)
CHLOR = forms typical of Chloridoideae (C_4)
PACMAD general = morphotypes found in C_3/C_4 open-habitat grasses in the PACMAD clade
OTHG = other (non-diagnostic, unknown, and unidentifiable) Poaceae GSSCs
AQ = phytoliths from wetland plants, including an unknown aquatic monocotyledon
NDG = non-diagnostic, potential grass phytoliths
NDO = non-diagnostic and unclassified phytoliths
*morphotypes named by ICPN Working Group 2005.
Morphotypes of **bold font** are the classification modification that used in this study.

Supplementary Table 2. Assemblage data and estimation of vegetation for phytolith assemblages extracted from the profile.

				GSSG																		
		FI-TOT					PACMAD TOT												Tree cover		Potential C_4	
Sample	Age (Ma)	Palm	Other FI	CH TOT	POOID-D	POOID-ND	PAN	CHLOR	PACMAD-general	OTHG	AQ	NDG	NDO	Diatoms	Sponge spicules	Charcoal >10μm	Total phytolith count	FI TOT+GSSC count	FI-t ratio	FI-t ratio 95% C.I.	PAN+CHLOR (min estimation)	PACMAD (max estimation)
Q4-0	0.01	0	0	0	6.52	8.35	0.51	0.07	0	0.37	0.22	70.1	13.9	6	0	944	1366	216	0.00%	0.00%	3.79%	3.79%
Q4-20	0.01	0	0	0	8.18	13	0.44	0.09	0	0.44	0	56.5	21.3	0	0	330	1125	249	0.00%	0.00%	2.46%	2.46%
Q4-40	0.01	0	0	0	3.89	12.3	0.16	0	0	0.4	0	61.8	21.5	0	6	183	1261	211	0.00%	0.00%	0.97%	0.97%
Q4-60	0.01	0	0	0	1.06	6.19	0.08	0	0	0.2	0	75.2	17.2	0	2	248	2454	185	0.00%	0.00%	1.11%	1.11%
Q4-80	0.01	0	0	0.09	1.68	6.16	0	0	0	0.47	0	75.4	16.2	0	0	342	1072	90	0.00%	0.00%	0.00%	0.00%
Q4-100	0.02	0	0	0	1.24	4.43	0.26	0.07	0	0.13	0	84.4	9.52	0	0	483	1534	94	0.00%	0.00%	5.43%	5.43%
Q4-120	0.04	0	0	0.12	0.5	4.21	0.37	0	0	0.99	0	79.8	14	0	1	546	807	50	0.00%	0.00%	7.14%	7.14%
Q4-140	0.06	0.15	0	0	0.91	6.35	0	0	0	0	0	77.2	15.4	0	0	277	661	49	2.04%	3.96%	0.00%	0.00%
Q4-160	0.08	0	0	0	0.94	5.62	0	0	0	0	0	76.8	16.6	0	0	359	427	28	0.00%	0.00%	0.00%	0.00%
Q4-180	0.10	0	0	0	0	2.77	0.33	0.16	0	0.16	0	75.7	20.8	0	0	740	614	21	0.00%	0.00%	15.00%	15.00%
MJP-S1-300	0.13	0	0	0	1.16	4.22	0	0	0	0.15	0	79.3	15.1	0	0	1013	687	38	0.00%	0.00%	0.00%	0.00%
MJP-S1-400	0.13	0	0	0	1.53	13	0	0	0	1.53	0	60.3	23.7	0	0	1455	131	21	0.00%	0.00%	0.00%	0.00%
2#SWC-550	2.90	0	0	0	39.7	3.82	7.63	0.76	23.7	3.82	0	9.92	10.7	2	0	6952	131	104	0.00%	0.00%	11.11%	42.42%
2#SWC-650	2.94	0	6.34	0	19.7	3.39	4.53	0.23	10.4	7.69	0	15.2	31.9	20	0	1904	442	231	12.12%	4.21%	10.92%	34.84%
3#SWC-950	3.80	0	0.11	0	35.8	0.67	0.55	0	2.89	0.11	0	41.1	18.8	10	0	3428	901	362	0.28%	0.54%	1.39%	8.59%
3#SWC-1850	4.15	0	0.88	4.42	1.77	57.5	3.54	0.88	11.5	1.77	0	9.73	7.96	4	0	6880	113	93	1.08%	2.10%	5.50%	19.78%
3#SWC-2250	4.29	0	0	1.27	7.01	10.2	8.92	0.64	37.6	3.82	0.64	12.1	17.8	14	0	6630	157	109	0.00%	0.00%	14.56%	71.84%
3#SWC-2650	4.90	0	2.87	0.82	11.1	0.82	17.2	2.87	45.5	1.64	0	9.02	8.2	11	0	1507	244	202	3.47%	2.52%	24.77%	80.87%

3#SWC-2750	5.00	0	0	0	4.83	7.59	17.9	1.38	53.1	6.9	0	2.76	5.52	50	0	2979	145	133	0.00%	0.00%	22.76%	85.37%
3#SWC-2850	5.04	0	0	1.98	4.95	10.9	16.8	0	40.6	8.91	0	10.9	4.95	22	1	3670	101	85	0.00%	0.00%	22.37%	76.32%
3#SWC-2950	5.08	0	0	0	4.4	6.08	15.1	7.34	55.6	3.35	0	3.98	4.19	150	0	1934	477	438	0.00%	0.00%	25.36%	88.15%
3#SWC-3250	5.21	0	0	0.53	2.66	9.04	17.6	2.66	38.8	8.51	0	7.45	12.8	66	1	8255	188	150	0.00%	0.00%	28.36%	82.84%
3#SWC-3650	5.74	0	0.65	0	10.5	7.84	21.6	1.96	26.8	5.23	0	8.5	17	112	0	5450	153	114	0.88%	1.71%	33.98%	72.69%
3#SWC-3750	5.89	0	2.35	0.43	14.5	4.9	13.2	1.28	22	3.62	0	21.5	16.2	102	0	3109	469	292	3.77%	2.18%	24.79%	62.33%
3#SWC-3850	6.05	0	0	0	7.87	5.38	14.3	2.48	50.7	1.04	0	8.49	9.73	194	0	3630	483	395	0.00%	0.00%	20.77%	83.59%
3#SWC-3950	6.14	0	0	0	4.72	4.72	12.3	17	50.9	5.66	0	4.72	0	21	0	2232	106	106	0.00%	0.00%	32.63%	89.47%
3#SWC-4050	6.23	0	0.29	1.45	6.69	9.3	11	0.29	50.3	1.45	0	10.8	8.43	26	0	7212	344	289	0.36%	0.69%	14.29%	77.66%
3#SWC-4350	6.40	0	0	0.99	10.9	7.92	23.8	1.98	12.9	3.96	0	19.8	17.8	15	0	9694	101	63	0.00%	0.00%	44.07%	66.10%
3#SWC-4450	6.49	0	0	0.4	9.92	6.75	13.9	13.9	27.8	5.56	0	10.3	11.5	190	0	14837	252	197	0.00%	0.00%	38.25%	76.50%
3#SWC-4550	6.67	0	0.43	0	13.5	2.8	15.3	3.44	41.9	2.58	0	8.6	11.4	195	0	6689	465	372	0.54%	0.74%	24.17%	78.35%
3#SWC-4950	7.19	0	0	0	12.9	8.63	11.5	2.16	43.2	4.32	0	11.5	5.76	253	0	7282	139	115	0.00%	0.00%	17.43%	72.48%
6#SWC-1850	8.00	0	2.62	2.33	7.87	2.04	5.83	2.62	23	3.5	0	39.9	10.2	6	0	2595	343	171	5.26%	3.35%	18.32%	68.21%
5#SWC-3650	9.88	0	0.29	0	3.71	6.57	9.14	0	28.6	13.1	0	16.6	22	7	0	2665	350	215	0.47%	0.91%	18.96%	78.21%
5#SWC-7350	10.49	0	0	0	9.69	3.39	16	4.36	57.4	2.42	0	2.66	4.12	17	0	1105	413	385	0.00%	0.00%	22.40%	85.60%
5#SWC-7650	10.55	0.17	0.17	0.17	20.5	15	14.6	10.7	32.2	2.41	0.17	2.07	1.89	31	0	1261	581	557	0.36%	0.50%	27.07%	61.52%
5#SWC-7750	10.57	0	0	0	12.2	9.25	25.1	9.64	39.2	1.03	0	1.54	2.06	107	0	807	778	750	0.00%	0.00%	36.39%	77.49%
5#SWC-7850	10.59	0	0	0	11.4	10.2	10	7.13	47.2	9.1	0	1.21	3.79	26	0	765	659	626	0.00%	0.00%	19.96%	74.91%
5#SWC-8650	10.77	0	0.69	0	13.1	4.14	5.52	2.07	8.97	5.52	0	37.9	22.1	49	0	2541	145	58	1.72%	3.35%	22.06%	48.14%

Q4, MJP from Loess; 2#SWC, 3#SWC from Lantian Formation; 6#SWC, 5#SWC from Bahe Formation.

Supplementary Table 3. Climate range of each subfamily of Poaceae in China

Subfamily	MAT (°C)	WMMT (°C)	CMMT (°C)	MAP (mm)	WMMP (mm)	CMMP (mm)	WSP (mm)	DT (°C)
Aristidoideae	2.0~24.0	10.9~28.6	-8.3~19.6	405~1672	46~128	2~59	334~1235	5.4~19.2
Arundinoideae	8.5~25.4	17.0~30.3	-1.9~19.5	706~2052	33~267	14~99	294~1408	8.3~24.4
Bambusoideae	7.9~25.4	16.0~27.7	-0.2~23.0	738~2847	76~420	13~225	385~2527	3.7~22.8
Chloridoideae	0.1~25.3	9.6~28.7	-18.1~22.3	273~2132	11~333	5~123	100~1489	4.5~37.1
Danthonioideae	14.4~17.7	20.5~26.9	8.6~8.7	225~981	6~53	41~101	47~391	11.8~18.3
Micrairoideae	15.5~24.0	22.5~27.3	5.4~19.6	1350~1966	148~225	39~85	822~1322	7.5~20.7
Oryzoideae	9.7~26.8	18.9~30.1	0.5~24.1	812~2410	51~199	36~149	417~1575	3.9~24.6
Panicoideae	3.3~26.8	13.8~29.6	-8.9~24.2	442~2694	43~370	6~205	263~1641	2.8~24.1
Pooideae	-13.6~15.3	3.5~26.4	-32.9~9.0	224~1674	9~257	3~120	96~1197	10.9~43.2

MAT: mean annual temperature, WMMT: warmest month mean temperature, CMMT: coldest month mean temperature, MAP: mean annual precipitation, WMMP: warmest month mean precipitation, CMMP: coldest month mean precipitation, WSP: warm season (April to September) precipitation, DT: difference in temperature of the warmest and coldest months.

Supplementary Table 4. Climate range of each subfamily of Poaceae in global scale

Subfamily	MAT (°C)	WMMT (°C)	CMMT (°C)	MAP (mm)	WMMP (mm)	CMMP (mm)	DT (°C)
Aristidoideae	2.1~28.4	10.8~33.2	-7.8~25.5	34~1966	0~276	0-144	0.9~29.0
Arundinoideae	5.3~25.4	15.5~30.2	-6.5~19.3	51~2069	3~266	1~160	4.2~24.3
Bambusoideae	6.9~27.2	10.5~30.2	-3.4~25.9	685~4902	22~501	4~535	0.7~25.0
Chloridoideae	0.1~29.0	9.3~34.2	-18.3~25.8	73~4093	0~437	0~362	0.8~37.1
Danthonioideae	3.3~21.0	10.1~26.8	-7.6~20.3	134~5927	5~542	8~523	1.0~26.7
Micrairoideae	8.4~27.5	18.1~31.5	-3.0~25.0	258~2885	26~355	1~230	1.1~21.4
Oryzoideae	5.6~27.8	13.7~31.2	-10.3~25.7	168~2717	6~275	6~236	2.0~30.0
Panicoideae	1.1~29.2	12.3~34.2	-11.9~35.9	102~3610	4~360	0~496	0.9~31.5
Pooideae	-18.3~22.5	3.3~30.7	-35.8~20.4	23~5518	0~502	1~482	0.8~47.5

The abbreviations are same to Supplementary Table 3.

Supplementary Table 5. Reconstructed climate parameters and the comparison with the modern climate of Weinan, calculated by the Coexistence Approach method and ecosystem matching

	Weather station (Weinan)	CA			CA and ecosystem matching		
		11.0~4.2 Ma	4.2~2.6 Ma	2.6~0 Ma	11.0~4.2 Ma	4.2~2.6 Ma	2.6~0 Ma
MAT (°C)	13.8	9.7~15.3	9.7~15.3	3.3~15.3	11~15.3	9.7~11	3.3~11
WMMT (°C)	26.8	18.9~26.4	18.9~26.4	13.8~26.4	20~26.4	18.9~20	13.8~20
CMMT (°C)	-0.3	0.5~9	0.5~9	-8.9~9	2~9	0.5~2	-8.9~2
MAP (mm)	569	812~1673	812~1673	441~1673	812~1673	812~900	441~900
WMMP (mm)	95	76~199	51~199	43~257	76~199	76~130	43~130
CMMP (mm)	6	36~120	36~120	6~120	36~120	36~74	6~74
WSP (mm)	439	417~1197	417~1197	265~1197	427~1197	417~540	263~540
DT (°C)	27.1	10~22.8	10~24.1	10~24.1	10~18	15~24.1	15~24.1

Climate data of Weinan from China meteorological data network [http://data.cma.cn], Time period of 198101~201012. The abbreviations are same to Supplementary Table 3.

Supplementary References

1. Wang, Y.J. & Lü, H.Y. *Phytolith study and its application*, 228 (China Ocean Press, Beijing, 1993).
2. Gu, Y.S., Zhao, Z.J. & Pearsall, D.M. Phytolith morphology research on wild and domesticated rice species in East Asia. *Quatern. Int.* **287**, 141-148 (2013).
3. Gu, Y.S., Liu, H.Y., Wang, H.L., Li, R.C. & Yu, J.X. Phytoliths as a method of identification for three genera of woody bamboos (Bambusoideae) in tropical southwest China. *J. Archaeol. Sci.* **68**, 46-53 (2016).
4. Prasad, V. *et al*. Late Cretaceous origin of the rice tribe provides evidence for early diversification in Poaceae. *Nat. Commun.* **2**, 480 (2011).
5. Edwards, E.J. & Smith, S.A. Phylogenetic analyses reveal the shady history of C_4 grasses. *Proc. Natl. Acad. Sci. USA.* **107**, 2532-2537 (2010).
6. Kriticos, D.J. *et al*. CliMond: global high-resolution historical and future scenario climate surfaces for bioclimatic modelling. *Methods Ecol. Evol.* **3**, 53-64 (2012).
7. Soreng, R.J. *et al*. A worldwide phylogenetic classification of the Poaceae (Gramineae). *J. Syst. Evol.* **53**, 117-137 (2015).
8. Soreng, R.J. *et al*. A worldwide phylogenetic classification of the Poaceae (Gramineae) II: An update and a comparison of two 2015 classifications. *J. Syst. Evol.* **55**, 259-290 (2017).
9. Ma, Y.Z., Li, J.J. & Fang, X.M. Pollen-spores in the red bed during 30.6－5.0 Ma in the Linxia Basin and climatic evolution. *Chinese science bulletin* **43**, 301-304 (1998).
10. Jiang, H.C. & Ding, Z.L. A 20 Ma pollen record of East-Asian summer monsoon evolution from Guyuan, Ningxia, China. *Palaeogeogr. Palaeoclimatol. Palaeoecol.* **265**, 30-38

(2008).

11. Tang, Z.H. *et al*. Late Cenozoic central Asian drying inferred from a palynological record from the northern Tian Shan. *Earth Planet. Sci. Lett.* **302**, 439-447 (2011).
12. Lu, H.Y., Wang, X.Y. & Li, L.P. Aeolian sediment evidence that global cooling has driven late Cenozoic stepwise aridification in central Asia. *Geological Society, London, Special Publications* **342**, 29-44 (2010).
13. Lu, H.Y. & Guo, Z.T. Evolution of the monsoon and dry climate in East Asia during late Cenozoic: A review. *Science. China Earth Sciences* **57**, 70-79 (2014).
14. Pagani, M., Zachos, J.C., Freeman, K.H., Tipple, B. & Bohaty, S. Marked decline in atmospheric carbon dioxide concentrations during the Paleogene. *Science* **309**, 600-603 (2005).
15. Seki, O. *et al*. Alkenone and boron-based Pliocene pCO_2 records. *Earth Planet. Sci. Lett.* **292**, 201-211 (2010).
16. Zhang, Y.G., Pagani, M., Liu, Z., Bohaty, S.M. & DeConto, R. A 40-million-year history of atmospheric CO_2. *Philosophical Transactions of the royal society A-Methematical, Physical and Engineering sciences* **371**(2013).
17. Badger, M.P., Schmidt, D.N., Mackensen, A. & Pancost, R.D. High-resolution alkenone palaeobarometry indicates relatively stable pCO_2 during the Pliocene (3.3-2.8 Ma). *Philos Trans A Math Phys Eng Sci* **371**, 20130094 (2013).
18. Bartoli, G., Hönisch, B. & Zeebe, R.E. Atmospheric CO_2 decline during the Pliocene intensification of Northern Hemisphere glaciations. *Paleoceanography* **26**(2011).
19. Martínez-Botí, M.A. *et al*. Plio-Pleistocene climate sensitivity evaluated using high-

resolution CO_2 records. *Nature* **518**, 49-54 (2015).

20. Stap, L.B. *et al*. CO_2 over the past 5 million years: Continuous simulation and new δ^{11}B-based proxy data. *Earth Planet. Sci. Lett.* **439**, 1-10 (2016).
21. Tripati, A.K., Roberts, C.D. & Eagle, R.A. Coupling of CO_2 and ice sheet stability over major climate transitions of the Last 20 Million years. *Science* **326**, 1394-1397 (2009).
22. Beerling, D.J. & Royer, D.L. Convergent Cenozoic CO_2 history. *Nat. Geosci.* **4**, 418-420 (2011).
23. Ogg, J.G. Geomagnetic Polarity Time Scale. in *The Geologic Time Scale 2012* (eds. Gradstein, F.M., Ogg, J.G., Schmitz, M.D. & Ogg, G.M.) 85-113 (Elsevier B.V., 2012).
24. Zheng, H.B., An, Z.S. & Shaw, J. New contributions to Chinese Plio-Pleistocene magnetostratigraphy. *Phys. Earth Planet. In.* **70**, 146-153 (1992).
25. An, Z.S. *et al*. Red clay sequences in Chinese Loess Plateau and recorded paleoclimate events of the late tertiary. *Quaternary Sciences* **05**, 435-446 (2000).
26. Wang, B. *et al*. Middle Miocene eolian sediments on the southern Chinese Loess Plateau dated by magnetostratigraphy. *Palaeogeogr. Palaeoclimatol. Palaeoecol.* **411**, 257-266 (2014).
27. Jiang, H.C. & Ding, Z.L. Spatial and temporal characteristics of Neogene palynoflora in China and its implication for the spread of steppe vegetation. *J. Arid Environ.* **73**, 765-772 (2009).
28. Qiu, Z.D. *et al*. Neogene Land Mammal Stages/Ages of China-Toward the Goal to Establish an Asian Land Mammal Stage/Age Scheme. in *Fossil Mammals of Asia-Neogene Biostratigraphy and Chronology* (eds. Wang, X.M., Flynn, L.J. & Fortelius, M.) 29-90

(Columbia University Press, New York, 2013).

29. Wang, Y. *et al*. Stable isotopes in fossil mammals, fish and shells from Kunlun Pass Basin, Tibetan Plateau: Paleo-climatic and paleo-elevation implications. *Earth Planet. Sci. Lett.* **270**, 73-85 (2008).

30. Zhang, C.F. *et al*. Diets and environments of late Cenozoic mammals in the Qaidam Basin, Tibetan Plateau: Evidence from stable isotopes. *Earth Planet. Sci. Lett.* **333-334**, 70-82 (2012).

31. Wang, Y. & Deng, T. A 25 m.y. isotopic record of paleodiet and environmental change from fossil mammals and paleosols from the NE margin of the Tibetan Plateau. *Earth Planet. Sci. Lett.* **236**, 322-338 (2005).

32. Arppe, L., Kaakinen, A., Passey, B.H., Zhang, Z. & Fortelius, M. Small mammal tooth enamel carbon isotope record of C_4 grasses in late Neogene China. *Global Planet. Change* **133**, 288-297 (2015).

33. Deng, T., Dong, J.S. & Wang, Y. The evidence of fossil carbon isotopes of the climatic event at the beginning of Quaternary. *Chinese Science Bulletin* **44**, 477-480 (1999).

34. Passey, B.H. *et al*. Strengthened East Asian summer monsoons during a period of high-latitude warmth? Isotopic evidence from Mio-Pliocene fossil mammals and soil carbonates from northern China. *Earth Planet. Sci. Lett.* **277**, 443-452 (2009).

35. Zhang, C.F., Wang, Y. & Deng, T. C_4 expansion in the central Inner Mongolia during the latest Miocene and early Pliocene. *Earth Planet. Sci. Lett.* **287**, 311-319 (2009).

36. Gaboardi, M., Deng, T. & Wang, Y. Middle Pleistocene climate and habitat change at Zhoukoudian, China, from the carbon and oxygen isotopic record from herbivore tooth

enamel. *Quaternary Res.* **63**, 329-338 (2005).

37. Still, C.J., Berry, J.A., Collatz, G.J. & DeFries, R.S. Global distribution of C_3 and C_4 vegetation: Carbon cycle implications. *Global Biogeochem. Cy.* **17**, 6-1-6-14 (2003).

38. Lü, H.Y. *et al*. Phytoliths as quantitative indicators for the reconstruction of past environmental conditions in China I: phytolith-based transfer functions. *Quaternary Sci. Rev.* **25**, 945-959 (2006).

39. Strömberg, C.A.E. & McInerney, F.A. The Neogene transition from C_3 to C_4 grasslands in North America: assemblage analysis of fossil phytoliths. *Paleobiology* **37**, 50-71 (2011).
